# Distinct HLA Associations for Antibody Multireactivity With Citrulline‐Containing Type II Collagen Epitopes Versus More Limited Antibody Reactivity With Citrulline‐Containing IgG Epitopes in Rheumatoid Arthritis

**DOI:** 10.1002/art.43424

**Published:** 2026-01-11

**Authors:** S. Janna Bashar, Courtney B. Myhr, Adam H. Titi, Zihao Zheng, Miriam A. Shelef

**Affiliations:** ^1^ Department of Medicine University of Wisconsin–Madison; ^2^ Department of Statistics University of Wisconsin–Madison; ^3^ William S. Middleton Memorial Veterans Hospital Madison Wisconsin

## Abstract

**Objective:**

Anticitrullinated protein antibodies (ACPAs) in rheumatoid arthritis (RA) can be promiscuous, with cross‐reactive binding to many antigens containing short motifs, or private with little cross‐reactivity. Also, ACPA reactivity patterns differ among patients with RA, including for motif‐containing epitopes in important self‐antigens like collagen and IgG (bound by RA‐associated rheumatoid factors [RFs]), with limited understanding of the underlying mechanism. The objective of this study was to determine if HLA alleles associate with ACPA reactivity patterns.

**Methods:**

For 100 ACPA+RF+ participants with RA, serum IgG binding was quantified by enzyme‐linked immunosorbent assay to 10 citrulline‐containing peptides derived from Type II collagen and IgG1 (nine with motifs), and HLA loci were genotyped. Also, antibody and serum multireactivity were evaluated. HLA alleles present differentially in RA participants with high versus low IgG binding to specific peptides, as well as with multireactivity versus limited reactivity were identified by Fisher's exact test.

**Results:**

Serum IgG multireactivity for citrulline‐glycine motif‐containing collagen peptides was high, at least partially due to promiscuous antibodies. HLA‐DQA1*01:02 was present in more participants with anticitrullinated collagen antibodies and multireactive sera. In contrast, serum multireactivity was low for IgG1‐derived peptides due at least in part to more private antibodies. Shared epitope–containing HLA‐DRB1*04:01 was present more frequently in participants with RA‐associated RFs irrespective of the citrulline‐serine motif and less frequently in participants with anticitrullinated collagen antibodies. Several HLA alleles associated with specific antibody reactivities.

**Conclusion:**

Different HLA alleles may contribute to the different reactivity patterns of promiscuous anticitrullinated collagen antibodies and more private RA‐associated RFs.

## INTRODUCTION

Anticitrullinated protein antibodies (ACPAs) are highly specific markers of rheumatoid arthritis (RA), a chronic autoimmune and inflammatory disease. Since their initial discovery over two decades ago,^1^ much has been learned about how ACPAs bind an enormous repertoire of antigens. Many ACPAs are actually multireactive antimodified protein antibodies that can recognize thousands of citrullinated (arginines converted to citrullines), homocitrullinated (lysines converted to homocitrullines), and other antigens.^2^ The multireactivity of at least some ACPAs appears to be due to the recognition of very short motifs like citrulline‐glycine, irrespective of protein identity.^2^ In contrast, other ACPAs are more selective and bind a limited number of antigens, leading to the labels “promiscuous” and “private.”^3^ These different ACPAs bind a variety of proteins, such as fibrin,^4^ histones,^5^ and vimentin,^6^ some with important roles in RA pathogenesis, in either a specific or nonspecific manner. The factors that underlie the development of different ACPAs are still emerging yet are central to understanding RA pathophysiology.

One important autoantibody target in RA is Type II collagen, an articular cartilage protein. Injection of Type II collagen with adjuvant drives inflammatory arthritis in mice,^7^ and anticollagen antibodies induce arthritis in naive mice.^8^ Antibodies against native Type II collagen are also found in human RA,^9^ and at least some of those anticollagen antibodies bind the same conformational native epitope as a pathogenic murine anticollagen antibody.^10^ People with RA also generate autoantibodies against citrullinated collagen, even more so than against native collagen.^11^ Interestingly, IgG in RA can recognize cyclic citrulline–containing collagen‐derived peptides, particularly when a citrulline‐glycine multireactivity motif is present,^12^ whereas murine anticollagen antibodies bind to conformational native epitopes.^13^ Of note, RA antibodies that react with cyclic citrulline–containing collagen peptides also bind to arthritic joints.^14^ However, not everyone with RA generates these antibodies, and there is a wide variety of IgG‐binding patterns to different collagen peptides, even when citrulline‐glycine is present.^12^ The mechanisms underlying these differences are poorly understood.

IgG is another important autoantibody target in RA. Rheumatoid factors (RFs), which recognize the Fc region of IgG, bind ACPA immune complexes to enhance macrophage‐mediated inflammation^15^ and complement‐mediated inflammation.^16^ RFs are canonically known to bind two conformational epitopes of IgG,^17^, ^18^ but RFs also have unique disease‐specific IgG reactivities and multireactivities.^19^, ^20^, ^21^, ^22^ For example, whereas COVID‐19–associated RFs bind IgG and pathogen epitopes based upon an aspartic acid–dominated tripeptide motif, RA‐associated RFs, whose reactivity overlaps with multireactive ACPAs, bind citrulline‐containing and homocitrulline‐containing linear IgG epitopes, potentially due in part to a citrulline‐serine motif.^20^, ^21^, ^22^, ^23^ Like promiscuous and private ACPAs, RFs have varying degrees of multireactivity.^24^ Also, similar to anticitrullinated collagen antibodies, RA sera have differing patterns of RA‐associated RF binding to IgG epitopes despite the presence of citrulline‐serine,^20^, ^21^, ^22^, ^23^ with no clear underlying mechanism.

One factor that may underlie the development of antibodies with different reactivities is the HLA. RA risk and ACPAs are most famously linked to HLA alleles with the shared epitope, a five amino acid consensus sequence found in HLA‐DRB1.^25^, ^26^ Shared epitope–containing HLAs have been reported to have preferential affinity for citrulline‐containing peptides, although variability exists among HLAs and peptides.^27^, ^28^, ^29^, ^30^, ^31^ Also, HLA alleles without the shared epitope, including HLA‐DQ and HLA‐DP alleles, as well as amino acids outside of the shared epitope region have been associated with RA, ACPAs, and the presentation of citrulline‐containing peptides.^32^, ^33^, ^34^, ^35^, ^36^

Some work has been done to evaluate HLA alleles in the context of specific antibodies as well. Because RA‐associated RFs are newly identified, no HLA studies have been performed. However, antibodies against Type II collagen were found to be associated with HLA‐DRB1*01 and HLA‐DRB1*03, but the specific anticollagen antibodies and the specific HLA alleles are unknown.^37^ Also, HLA‐DRB1*04 was found to be associated with private antibodies against a native Type II collagen triple helical peptide, a citrulline‐containing α‐enolase peptide, and citrullinated fibrinogen.^38^ However, neither specific HLA alleles nor multireactive antibodies were described. These findings provide evidence of a role for HLA in shaping the RA autoantibody repertoire, but mysteries remain regarding how HLA alleles relate to specific antibodies and multireactivity. Thus, the objective of this study was to determine if specific HLA alleles associate with specific anticitrullinated collagen antibodies, RA‐associated RFs, and/or multireactivity to better understand the development of pathogenic antibodies in RA.

## MATERIALS AND METHODS

### Human subjects

Human subjects research was approved by the University of Wisconsin Institutional Review Board and was conducted in compliance with the Declaration of Helsinki. All human subjects provided written informed consent. Sera and DNA from subjects diagnosed with RA by a rheumatologist and with anti–cyclic citrullinated peptide and RF levels more than two times the upper limit of normal as well as sera from control subjects without known autoimmune or inflammatory disease as previously defined were obtained from the University of Wisconsin Rheumatology Biorepository.^39^, ^40^ Participants with RA are described in Supplementary Table 1.

### Enzyme‐linked immunosorbent assay

Enzyme‐linked immunosorbent assay (ELISA) was performed as previously described^21^ with minor modifications. ELISA plates were incubated with a 0.1 μM concentration of peptides (Supplementary Table 2), incubated with sera diluted at 1:500 for collagen Type II α1 (COL2A1) and 1:200 for IgG1 peptides, and IgG was detected with mouse anti‐human IgG (clone JDC‐10, SouthernBiotech). RA sera with IgG binding values >1 SD above mean control IgG binding to each citrulline‐containing peptide (marked with a B to represent the amino acid citrulline) were defined as “high binding” or “positive” versus all other sera defined as “low binding” or “negative.”

### HLA genotyping

Genotyping of HLA‐DPA1, HLA‐DPB1, HLA‐DQA1, HLA‐DQB1, HLA‐DRB1, and HLA‐DRB345 alleles was performed using next‐generation sequencing by CD Genomics.

### Antibody purification

Antibodies that bind to COL2A1‐885B and IgG1‐289B were purified and used in ELISA as previously,^20^ with modifications. Briefly, streptavidin‐sepharose beads (Abcam, Waltham, MA) were incubated with an equal volume of 0.02 mM biotinylated peptide, washed, and loaded onto a column. Sera diluted 1:4 were added and incubated overnight at 4°C; columns were then washed, and antibody was eluted from the beads and concentrated using Amicon Ultra 30 kDa molecular weight cut‐off filters (MilliporeSigma, Burlington, MA). Antibody concentration was determined by ELISA using standard curves generated from purified IgG (Novus Biologicals, Centennial, CO). Purified antibodies were tested at 20 ng/mL to determine multireactivity.

### Statistics

IgG‐binding levels were compared by Kruskal‐Wallis test with Dunn's multiple comparisons test for more than two groups and a Mann‐Whitney test for two groups. The frequency of the presence of at least one copy of each unique HLA allele was compared for high and low binding groups by Fisher's exact test. All analyses were performed using Prism (GraphPad Software, San Diego, CA), and *P* < 0.05 was considered significant.

## RESULTS

We began by evaluating IgG binding in seropositive RA to all possible 12 amino acid linear peptides from COL2A1 in native, citrulline‐containing, and homocitrulline‐containing forms using data from two previously published high‐density peptide arrays.^23^, ^41^ We observed very high binding to many citrulline‐containing peptides and more modest binding to homocitrulline‐containing peptides (Supplementary Figure 1), consistent with previous results for these subjects and linear peptides derived from other proteins.^21^, ^23^ Also, the highest bound peptides overwhelmingly contained citrulline‐glycine, suggesting that this multireactivity motif is a major factor in anticitrullinated collagen reactivity.

**Figure 1:**
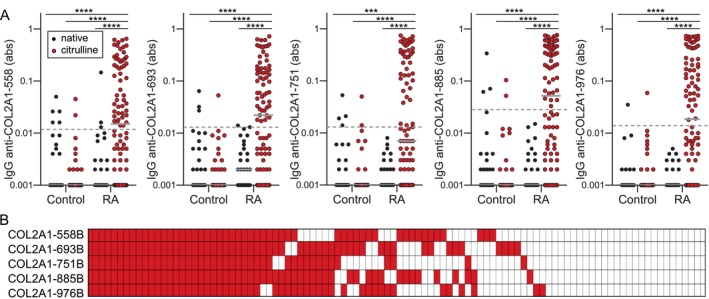
High, yet variable, IgG binding to citrulline‐containing Type II collagen‐derived linear peptides in RA. (A) IgG binding measured by enzyme‐linked immunosorbent assay for RA (n = 32) and control sera (n = 32) to five native linear COL2A1 peptides and IgG binding of control sera (n = 32) to the citrulline‐containing form of the peptides were compared to IgG binding for RA sera (n = 100) to the citrulline‐containing peptides by Kruskal‐Wallis with Dunn's multiple comparisons test (***P < 0.001; ****P < 0.0001; gray bars indicate medians; dashed lines indicate cut‐offs for high IgG binding 1 SD above the mean for control IgG binding to citrulline‐containing peptides). (B) Heatmap of positive binding (red) for each participant with RA (column) and peptide (row). abs, absorbance; B, citrulline; COL2A1, collagen Type II α1; RA, rheumatoid arthritis Color figure can be viewed in the online issue, which is available at http://onlinelibrary.wiley.com/doi/10.1002/art.43424/abstract.

To better understand the differential reactivities of potentially pathogenic anticollagen antibodies that target citrulline‐glycine, we honed in on five linear COL2A1 peptides that contained citrulline‐glycine, were highly bound by RA IgG in the array experiments, and had homologous epitopes in mice bound by arthritogenic autoantibodies when the peptides were in their triple helical form.^10^, ^13^, ^42^ In ELISA, we saw, as expected, significantly higher IgG binding in RA to the citrulline‐containing form than the native form of all five COL2A1 peptides, as well as higher binding in RA than in controls (Figure 1A). Also, similar to antibody binding to cyclic citrulline–containing collagen‐derived peptides,^12^ different RA sera demonstrated different degrees and patterns of IgG reactivity to the linear citrulline‐containing peptides despite the presence of citrulline‐glycine (Figure 1B). However, in general, serum multireactivity was high with >50% of sera with reactivity to any collagen peptide demonstrating reactivity against all five peptides (Supplementary Figure 2). Sera with anti–COL2A1‐751B antibodies had the highest level of multireactivity. Among sera with these antibodies, 88% had IgG that bound four or five peptides.

**Figure 2:**
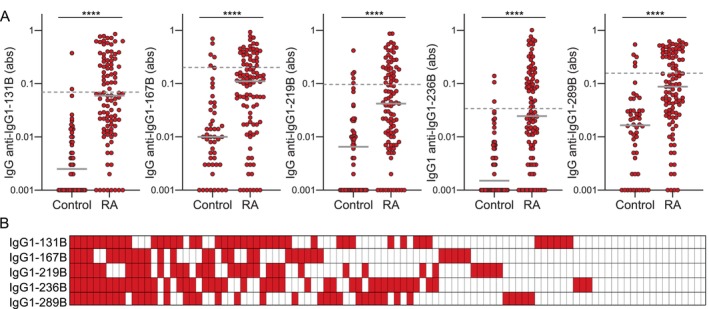
Increased, but variable, IgG binding to citrulline‐containing IgG1 peptides in RA. (A) IgG binding measured by enzyme‐linked immunosorbent assay for control (n = 50) versus RA sera (n = 100) to citrulline‐containing IgG1‐derived peptides was evaluated by Mann‐Whitney test (****P < 0.0001; gray bars indicate medians; dashed lines indicate cut‐offs for high IgG binding 1 SD above the mean for control IgG binding). (B) Heatmap of positive binding (red) for each participant with RA (column) and peptide (row). abs, absorbance; B, citrulline; RA, rheumatoid arthritis. Color figure can be viewed in the online issue, which is available at http://onlinelibrary.wiley.com/doi/10.1002/art.43424/abstract.

We also evaluated IgG binding to five IgG1‐derived citrulline‐containing peptides that are known to be bound at high levels in seropositive RA, four of which have citrulline‐serine.^20^, ^21^, ^22^ As with COL2A1, RA sera had significantly more binding to citrulline‐containing IgG1 peptides than control sera, with variability among sera in both the degree and pattern of reactivity (Figure 2). In contrast to COL2A1, serum multireactivity was low for the citrulline‐containing IgG1‐derived peptides (Supplementary Figure 2). Among sera with IgG that bound any IgG1 peptide, only approximately 10% had IgG that bound all five IgG1 peptides and approximately one‐third had IgG that bound four to five peptides. Sera with any reactivity with IgG1‐derived peptides most commonly bound two or three IgG1 peptides, as opposed to five peptides for collagen. However, it should be noted that the higher and more variable RA‐associated RF levels in controls raises the cut‐off for positivity in RA, which may underestimate RA‐associated RF presence in our cohort.

**Figure 3:**
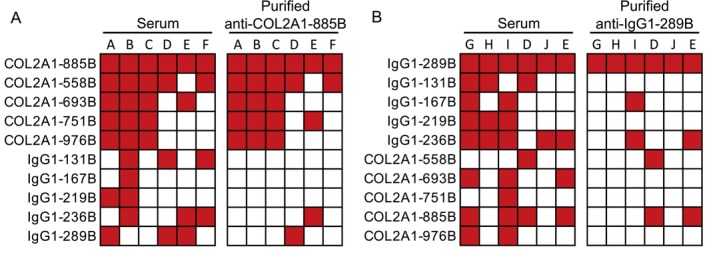
Rheumatoid arthritis antibody and serum multireactivity for Type II collagen‐ and IgG1‐derived peptides. (A) Antibodies that bind the COL2A1‐885B peptide were purified from three sera multireactive for all five collagen peptides and three sera with IgG reactivity to only two collagen peptides. IgG anti–COL2A1‐885B binding to the five collagen and five IgG peptides was quantified by enzyme‐linked immunosorbent assay. (B) Antibodies that bind to the IgG1‐289B peptide were purified from sera with reactivity to four or five IgG1 peptides or reactivity to two IgG1 peptides and then tested in enzyme‐linked immunosorbent assay for binding to all 10 peptides as in (A). Letters indicate specific participants. Positive results for serum reactivity according to previously noted cut‐offs, and binding values >5% of the peptide used to purify the tested antibody are depicted in red. COL2A1, collagen Type II α1. Color figure can be viewed in the online issue, which is available at http://onlinelibrary.wiley.com/doi/10.1002/art.43424/abstract.

Nonetheless, given the differences in multireactivity among sera for collagen‐ and IgG1‐derived peptides, we evaluated antibody promiscuity. Antibodies that bind COL2A1‐885B were elevated in all 40 of the RA sera with reactivity to at least four collagen peptides as well as sera with more limited reactivity. Also, COL2A1‐885B has only one citrulline and thus likely only one epitope recognized by purified ACPAs. Therefore, we purified antibodies that bound to COL2A1‐885B from three subjects with reactivity to all 5 peptides and three subjects with reactivity to 2 peptides and evaluated IgG binding of the anti‐COL2A1‐885B antibodies to all 10 COL2A1 and IgG1 peptides. As shown in Figure 3A, anti–COL2A1‐885B antibodies from multireactive sera bound all five collagen peptides but not any IgG1 peptides despite serum multireactivity with IgG1‐derived peptides. The binding of anti–COL2A1‐885B antibodies from limited reactivity sera primarily mirrored serum reactivity but not quite as closely as for the multireactive sera.

We performed similar experiments evaluating multireactivity with IgG1 peptides (Figure 3B). As noted above, serum multireactivity was low for IgG1‐derived peptides. However, anti–IgG1‐289B antibodies were present in all but 1 of the 13 RA serum samples with multireactivity to four or five peptides. Moreover, anti–IgG1‐289B antibodies were present at sufficiently high levels for antibody purification experiments, and IgG1‐289B contains only a single citrulline, a combination of features not present for the other four peptides. Thus, we purified anti–IgG1‐289B antibodies from sera that bound 4 to 5 IgG1 peptides or 2 IgG1 peptides and evaluated IgG binding to all 10 peptides. In contrast to the anti–COL2A1‐885B results, anti–IgG1‐289B antibodies purified from two of three multireactive sera had no cross‐reactivity with the other peptides. Anti–IgG1‐289B antibodies purified from limited reactivity sera more closely mirrored serum reactivity. Taken together, these data suggest that promiscuous antibodies contribute to high serum multireactivity with citrulline‐glycine–containing collagen‐derived peptides, whereas multiple, more private antibodies may underly the low serum multireactivity to IgG1‐derived peptides.

We then evaluated if the presence of IgG that binds to specific peptides might correlate with specific HLA alleles. We genotyped the HLA loci of the participants with RA and then determined if specific alleles were more highly represented in participants with RA with high or low binding for each peptide. For collagen (Table 1), we identified DQA1*01:02 as present in a higher percentage of RA participants with high IgG binding to COL2A1‐751B versus low IgG binding to that peptide. In contrast, DRB3*02:02, DQA1*05:05, and shared epitope–containing DRB1*04:01 were present in more RA participants with low IgG binding to COL2A1‐885B, COL2A1‐976B, and COL2A1‐693B, respectively.

**Table 1:** Association of HLA alleles with IgG that binds specific Type II collagen peptides*

Peptide	HLA allele	Allele frequency in high binders	Allele frequency in low binders	*P* value^a^
COL2A1‐693B, n/N (%)	DRB1*04:01	19/55 (35)	25/45 (56)	0.044
COL2A1‐751B, n/N (%)	DQA1*01:02	18/43 (42)	12/57 (21)	0.029
COL2A1‐885B, n/N (%)	DRB3*02:02	1/54 (2)	6/46 (13)	0.046
COL2A1‐976B, n/N (%)	DQA1*05:05	1/51 (2)	7/49 (14)	0.029

* COL2A1, collagen Type II α1.

^a^ Fisher's exact test.

For IgG binding to IgG1‐derived peptides (Table 2), we found that shared epitope–containing DRB1*04:01 is present in more RA participants with high IgG binding to IgG1‐167B, whereas DPB1*03:01 is present in more participants with low binding to that peptide. Also, DQA1*01:05 and shared epitope–containing DRB1*10:01 were present more frequently in high IgG‐binding subjects, and DPB1*04:02 in more low IgG‐binding subjects for IgG1‐219B. However, all participants who had DRB1*10:01 also had DQA1*01:05 and vice versa, making differentiating between those two alleles for analysis impossible.

**Table 2:** Association of HLA alleles with IgG that binds specific IgG1 peptides

Peptide	HLA allele	Allele frequency in high binders	Allele frequency in low binders	*P* value^a^
IgG1‐167B, n/N (%)	DRB1*04:01	19/30 (63)	25/70 (36)	0.015
	DPB1*03:01	1/30 (3)	14/70 (20)	0.035
IgG1‐219B, n/N (%)	DPB1*04:02	5/35 (14)	23/65 (35)	0.035
	DQA1*01:05^b^	4/35 (11)	0/65 (0)	0.013
	DRB1*10:01^b^	4/35 (11)	0/65 (0)	0.013

^a^ Fisher's exact test.

^b^ The same four subjects possessed both of these alleles.

**Figure 4:**
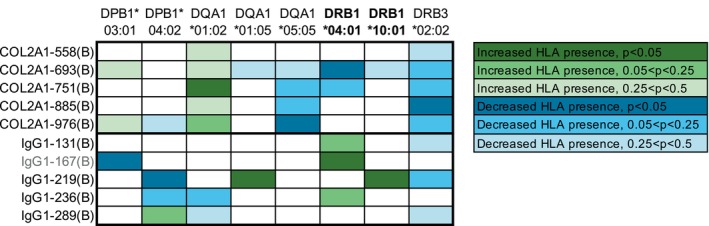
Heatmap of HLA presence in participants with rheumatoid arthritis with IgG binding to citrulline‐containing collagen and IgG1 peptides. For each HLA allele significantly associated with an antipeptide antibody in participants with rheumatoid arthritis, we depicted in a heatmap the association of that HLA allele with all 10 antibodies. Cells are colored different shades of green (HLA allele present more frequently in subjects with the antibody versus without) or blue (HLA allele present less frequently in subjects with the antibody versus without) based on the *P* value in a Fisher's exact test. White cells represent *P* ≥ 0.5. HLA alleles in bold contain the shared epitope, and peptide in gray has neither citrulline‐serine or citrulline‐glycine. COL2A1, collagen Type II α1.

Given the multireactivity observed for collagen peptides, we were surprised that each HLA variant associated with antibodies for only a single collagen peptide, although the only positive association (DQA1*01:02; Table 1) was with the antibody that had the highest level of serum multireactivity (IgG anti–COL2A1‐751B; Supplementary Figure 2). Because our sample size was only 100, we hypothesized that patterns of associations might be revealed if we looked at trends, not just significant *P* values. So, for each HLA variant significantly associated with antibodies that recognized any peptide, we created a heatmap of association trends for all 10 peptides. As shown in Figure 4, participants with IgG that binds any of the five collagen peptides show at least a trend toward more frequent presence of DQA1*01:02 and less frequent presence of DRB3*02:02. DQA1*05:05 had similar results as DRB3*02:02 but for only four of five peptides. Less uniformity was seen for the IgG1‐derived peptides, with no HLA demonstrating even a trend toward association with antibodies against all five peptides. However, DRB1*04:01 had at least a trend toward increased presence in participants with IgG that binds IgG1‐131B, IgG1‐167B, or IgG1‐236B. Combined with the positive association of DRB1*10:01 with IgG anti–IgG1‐219B, at least a trend toward more frequent presence of shared epitope–containing HLA exists for participants with RA‐associated RFs. Of note, the negative association of DRB1*04:01 with IgG that binds citrulline‐containing collagen peptides was not uniform across citrulline‐glycine–containing peptides.

Next, we evaluated the relationship between HLA and serum multireactivity. We compared HLA presence for participants with IgG binding to multiple peptides versus limited or no binding. For the collagen peptides, DQA1*01:02 was present in 43% of the 40 participants with RA with multireactivity across four to five peptides versus 22% of the 60 subjects with limited or no reactivity (0–3 peptides; *P* = 0.044). Moreover, all multireactive sera bound COL2A1‐885B, and DQA1*01:02 was present in 43% of those 40 participants compared to only 14% of the 14 participants with anti–COL2A1‐885B and limited reactivity (1–3 peptides, *P* = 0.058). For IgG1 peptides, only 13 participants had multireactivity across four to five peptides, which limited analysis. However, 34 participants had high IgG binding to three to five IgG1 peptides, and DRB1*04:01 was more frequent in these participants than in participants with binding to zero to two peptides (59% versus 36%; *P* = 0.036). These HLA associations with multireactivity are consistent with the peptide‐specific associations depicted in Figure 4. Together, these data suggest that multireactivity to citrulline‐glycine–containing collagen peptides is associated with HLA‐DQA1*01:02, whereas reactivity with some IgG1 peptides, with or without citrulline‐serine, is associated with HLA‐DRB1*04:01.

One factor to consider regarding these results is the high level of linkage disequilibrium among HLA alleles. Therefore, for HLA alleles commonly in linkage disequilibrium^43^ with DRB1*04:01 or DQA1*01:02 that were present in more than one participant in our cohort, we included the *P* values (all >0.05) for the above analyses (Supplementary Table 3). We also evaluated the association for each partial haplotype (ie, the combination of the allele of interest and the allele in linkage disequilibrium) for each antibody and multireactivity (Supplementary Table 4). The only statistically significant results were reduced presence of DRB1*04:01/DQB1*03:02 in participants with versus without IgG anti–COL2A1‐693B (20% versus 40%; *P* = 0.045), IgG anti–COL2A1‐976B (20% versus 39%; *P* = 0.047), and anticollagen multireactivity (18% versus 37%; *P* = 0.045). These findings are consistent with the negative association of DRB1*04:01 with IgG anti–COL2A1‐693B (Table 1) and the trend toward a negative association of DRB1*04:01 with anticollagen multireactivity (Supplementary Table 3; DRB1*04:01 present in 33% of people with versus 52% of people without anticollagen multireactivity; *P* = 0.067).

Finally, intrigued by the opposite relationship of DRB1*04:01 with antibodies that recognize IgG1 versus collagen peptides, we divided participants into four groups: multireactivity for collagen and IgG1 peptides, multireactivity for collagen only, multireactivity for IgG1 only, and no multireactivity (Supplementary Figure 3A). We then compared the presence of each allele in a pairwise manner using a Fisher's exact test. The only significant associations were found for DRB1*04:01, potentially due to the smaller sample sizes that result from dividing participants into four groups, with the strongest trend seen for DQA1*01:02. As shown in Supplementary Figure 3B, DRB1*04:01 was present in 74% of participants with multireactivity for IgG1 and not collagen, the two conditions positively associated with DRB1*04:01, compared with 28% of participants with multireactivity for collagen and not IgG1, the two conditions negatively associated with DRB1*04:01 (*P* = 0.006). The difference was less dramatic when comparing the presence of DRB1*04:01 in participants with multireactivity for IgG1 and not collagen versus participants with only one condition positively associated with DRB1*04:01 (ie, no multireactivity; 74% versus 41%; *P* = 0.027) or multireactivity with both peptides sets (74% versus 40%; *P* = 0.080). In contrast, DQA1*01:02 strongly trended toward greater presence in participants with multireactivity to collagen only as compared with subjects without collagen multireactivity either with (48% versus 16%; *P* = 0.052) or without (48% versus 24%; *P* = 0.062) IgG1 multireactivity. Together, these data re‐enforce the positive association of DRB1*04:01 with both the presence of IgG1 multireactivity and the absence of collagen multireactivity as compared with the positive association of DQA1*01:02 with collagen multireactivity independent from IgG1 multireactivity.

## DISCUSSION

This study aimed to determine if specific HLA alleles associate with specific anticitrullinated collagen antibodies, RA‐associated RFs, and/or multireactivity in RA. We discovered a novel positive association between DQA1*01:02 and multireactivity with citrulline‐glycine–containing Type II collagen‐derived peptides. Further, we found a positive association of shared epitope–containing DRB1*04:01 with antibodies that bind citrulline‐containing IgG epitopes in a more limited manner and a negative association of DRB1*04:01 with antibodies that bind citrulline‐glycine–containing collagen peptides. We also identified several new HLA associations with specific antibodies.

To assess HLA associations, we first evaluated reactivity patterns of RA sera with collagen‐ and IgG1‐derived peptides. As expected, sera had a variety of IgG‐binding patterns across participants, even when citrulline‐glycine or citrulline‐serine motifs were present. However, serum multireactivity was more prominent for collagen than IgG1 peptides. Similarly, purified anti–COL2A1‐885B antibodies were promiscuous, and anti–IgG1‐289B antibodies were more private. The multireactivity against collagen peptides is likely due to the presence of citrulline‐glycine, not the collagen protein, because citrulline‐glycine is a motif for ACPA antigens based on a wide variety of peptides derived from many different proteins.^2^, ^23^, ^44^ Type II collagen has many citrulline‐glycine pairs, which may underlie its frequent targeting in RA. In contrast, citrulline‐serine was only identified in a single study, and it was a motif for RA serum IgG binding, not monoclonal ACPA binding.^23^ Thus, citrulline‐serine could be a motif for more private antibodies that, perhaps, have unique reactivities based on binding to amino acids that neighbor the motif. Additional studies are needed to evaluate citrulline‐serine, citrulline‐glycine, IgG1, and collagen more independently to draw definitive conclusions.

The differential multireactivity for antibodies that bind citrulline‐containing collagen and IgG epitopes is intriguing, especially in the context of RA development. Because antibodies that bind citrullinated collagen have a high likelihood of being promiscuous, the original tolerance‐breaking antigen is unknown. Because of molecular mimicry with Epstein‐Barr virus proteins, citrullinated Type II collagen could be an original antigen driving RA.^45^ Alternatively, tolerance could be lost at a mucosal surface,^46^ with promiscuous ACPAs developing during the preclinical ACPA expansion period,^47^ perhaps due to epitope spreading. The promiscuous ACPAs could then bind citrullinated collagen in a nonspecific, although potentially pathogenic, manner. In contrast, RA‐associated RFs appear to be more private, although not completely private given some cross‐reactivity (Figure 3) and the binding of some promiscuous ACPAs to citrulline‐containing and homocitrulline‐containing IgG epitopes.^21^ Given this level of privacy, the presence of IgG at sites of infection, IgG citrullination,^48^ and RF cross‐reactivity with pathogen antigens,^20^, ^49^ tolerance could be specifically lost for citrullinated IgG early on the path to RA.^50^ However, future studies evaluating these antibodies in the preclinical RA period would be needed to more definitely determine antigen chronology.

In addition to, or perhaps due to, the differences in multireactivity, we identified different HLA associations for anticitrullinated collagen antibodies and RA‐associated RFs. DQA1*01:02 was the only HLA allele positively associated with citrullinated collagen reactivity in our cohort. It was present in more RA participants with IgG binding to COL2A1‐751B (Table 1), the antibody with the most associated serum multireactivity (Supplementary Figure 2), with similar trends for the other collagen peptides (Figure 4). DQA1*01:02 was also present in more RA participants with serum multireactivity to collagen peptides. Finally, among RA participants with anti–COL2A1‐885B (whose anticollagen multireactivity mirrored that of serum; Figure 3), those with multireactive sera had a strong trend of more frequently having DQA1*01:02 than those with limited reactivity sera. Together, these data suggest, albeit to some extent indirectly, that DQA1*01:02 is associated with promiscuous IgG that binds to citrulline‐glycine–containing collagen peptides. To the best of our knowledge, these findings provide the first evidence for a role for DQA1*01:02 in RA as well as the first positive HLA association with multireactivity. Interestingly, DQA1*01:02 is associated with increased lupus risk in multiple populations,^51^ and lupus autoantibodies can be promiscuous,^52^ raising the possibility that DQA1*01:02 could contribute to the development of promiscuous antibodies across diseases.

As opposed to the increased presence of DQA1*01:02 in participants with RA with anticitrullinated collagen antibodies, there was a decreased presence of DRB3*02:02 and DQA1*05:05, most prominently in RA participants with anti–COL2A1‐885B and anti–COL2A1‐976B, respectively (Table 1), as well as anticollagen antibodies in general (Figure 4). Given these broad associations across citrulline‐glycine–containing collagen peptides, these HLAs may be negatively associated with multireactivity. Little is known about these two alleles in the context of RA, leaving many unanswered questions about how they could protect against the formation of promiscuous RA autoantibodies. Also, there was a decreased presence of shared epitope–containing DRB1*04:01 in RA participants with anti–COL2A1‐693B with a similar trend for anti–COL2A1‐751B. Consistent with these findings, there was a trend toward a negative association of DRB1*04:01 with anticollagen multireactivity and a significant negative association of the DRB1*04:01/DQB1*03:02 partial haplotype with IgG anti–COL2A1‐693B, IgG anti–COL2A1‐976B, and anticollagen multireactivity. Interestingly, DRB1*04 positively associates with the native form of a peptide almost identical to COL2A1‐558 (C^III^),^38^ suggesting different HLA associations for citrullinated versus native epitopes of Type II collagen.

As opposed to the negative association with anticitrullinated collagen antibodies, DRB1*04:01 was present more frequently in RA participants with IgG anti–IgG1‐167B (Table 2) with a similar trend for IgG1‐131B and IgG1‐236B (Figure 4). DRB1*04:01 was also present more frequently in RA participants with serum multireactivity for IgG1 peptides. However, multireactivity was low for RA‐associated RFs, and IgG1‐167B does not have citrulline‐serine. Thus, DQA1*01:02 may be associated with promiscuous citrulline‐glycine–reactive anticollagen antibodies, whereas DRB1*04:01 may be associated with a subset of more private RA‐associated RFs. Interestingly, another shared epitope–containing HLA, DRB1*10:01, was more frequent in RA participants with IgG anti–IgG1‐219B (although, as noted above, it is in complete linkage disequilibrium with DQA1*01:05, limiting the strength of this association). Nonetheless, shared epitope–containing HLA could be positively associated with private ACPAs, including RA‐associated RFs, in contrast to a negative association with promiscuous citrulline‐glycine–reactive anticollagen antibodies. In support of this conclusion, shared epitope–containing HLAs recently have been shown to associate with noncitrulline‐glycine motif ACPAs more than citrulline‐glycine motif ACPAs.^53^ Also consistent with this finding is the positive association of DRB1*04 with three private antibodies^38^ and the positive association of DRB1*04:01 with ACPAs that bind a citrulline‐containing fibrin peptide without citrulline‐glycine (SGIGTLDGF**B**H**B**HPD).^54^ These studies did not evaluate the HLA‐DQ locus, but future studies should.

Finally, DPB1*04:02 and DPB1*03:01 were present more commonly in participants with RA with low binding to IgG1‐219B and IgG1‐167B, respectively. How DPB1 alleles impact RA development is only emerging,^32^ and the relationship between DPB1*04:02 and DPB1*03:01 and RA‐associated RFs will require more exploration.

The mechanism underlying the associations of HLAs with autoantibodies is not completely clear but may be due to HLA‐binding affinities for antigens, as previously explored for shared epitope–containing alleles. The more frequent presence of DRB1*04:01 in participants with RA‐associated RFs, two that bind a peptide with citrulline‐serine and one that binds a peptide without a motif, is consistent with DRB1*04:01 binding a variety of peptides with different amino acids adjacent to citrulline.^29^, ^36^ DRB1*10:01, which was positively associated with anti–IgG1‐219B, has similar variability.^55^, ^56^ Moreover, the negative association of DRB1*04:01 with anti–COL2A1‐693B in our study is consistent with poor binding of DRB1*04:01 to a citrulline‐containing collagen peptide with five identical amino acids (**B**GFPG) as COL2A1‐693B.^56^ These different binding patterns may be explained by the shared epitope–defined P4 pocket contacting citrulline^28^ and the nonshared epitope residues of other pockets differentially driving affinity for the rest of the peptide antigen.^29^ Thus, differential HLA affinity may be driving the differential HLA associations with specific antibodies. Alternatively, the hapten carrier model, in which PAD4 (carrier) is targeted by T cells that help B cells reacting to citrullinated peptides (haptens),^57^ may be contributing to the development of private and/or promiscuous ACPAs.

There are several limitations to our study. Most importantly, our sample size is small, which prevents statistical significance when correcting for multiple comparisons. Also, our participants are all North American and 85% White (Supplementary Table 1). These limitations reduce the statistical power, robustness, and generalizability of our findings. To partially address the issue of statistical power, we provided information about potentially biologically relevant trends, although these findings must be interpreted with caution. Further, although we performed a partial analysis of haplotypes, the small sample size may have led us to underestimate the contributions from alleles in linkage disequilibrium. Also, we were unable to purify antibodies that bind all 10 peptides from all sera to comprehensively evaluate multireactivity, limiting the strength of our conclusions about HLA associations with multireactivity. Additionally, our studies did not include peptides with citrulline‐glycine or citrulline‐serine that were not collagen derived or IgG1 derived, respectively, or collagen peptides that did not contain citrulline‐glycine, preventing us from differentiating between findings due to motifs versus specific proteins. Peptides also represented only linear, and not conformational, epitopes. Finally, we only demonstrated HLA associations with antibodies; thus, mechanisms remain unknown.

Nonetheless, this study identified a novel positive association for DQA1*01:02 and a negative association for DRB1*04:01 with promiscuous antibodies that bind citrulline‐glycine–containing Type II collagen‐derived peptides in RA as well as a positive association between shared epitope–containing HLA alleles, most prominently DRB1*04:01, and RA‐associated RFs, which appear to be more private. Future studies evaluating additional peptides from other important antigens as well as larger, more diverse cohorts are needed to further refine these associations as well as to define the underlying mechanisms.

## AUTHOR CONTRIBUTIONS

All authors contributed to at least one of the following manuscript preparation roles: conceptualization AND/OR methodology, software, investigation, formal analysis, data curation, visualization, and validation AND drafting or reviewing/editing the final draft. As corresponding author, Dr Shelef confirms that all authors have provided the final approval of the version to be published, and takes responsibility for the affirmations regarding article submission (eg, not under consideration by another journal), the integrity of the data presented, and the statements regarding compliance with institutional review board/Declaration of Helsinki requirements.

## Supporting information


**Disclosure form**.

**Data S1:** Bashar HLA data.

**Appendix S1:** Supplementary Information.
