# Supplementary material for: Distinct HLA Associations for Antibody Multireactivity With Citrulline‐Containing Type II Collagen Epitopes Versus More Limited Antibody Reactivity With Citrulline‐Containing IgG Epitopes in Rheumatoid Arthritis
Source: Arthritis Rheumatol. 2026 Jan 11;78(3):603–12. doi: 10.1002/art.43424 (PMC12991920; doi:10.1002/art.43424)
Supplement: Supplementary file 3 — Appendix S1: Supplementary Information. [file ART-78-603-s003.pdf]

Supplementary Material for:

Title: HLA-DQA1*01:02 is associated with antibody multi-reactivity with citrulline-containing type II collagen epitopes while HLA-DRB1*04:01 is associated with more private antibody reactivity with citrulline-containing IgG epitopes in rheumatoid arthritis

Authors: S. Janna Bashar, Courtney B. Myhr, Adam H. Titi, Zihao Zheng, and Miriam A. Shelef

Supplementary Table 1. Characteristics of rheumatoid arthritis participants

Characteristic	Participants (n=100)
Age, mean years (range)	61 (21-85)
Sex, female (%)	67 (67)
Race/Ethnicity, number (%)*	
White	85 (85)
Black	3 (3)
Hispanic	5 (5)
Native American (NA)	0 (0)
Asian	0 (0)
Pacific Islander	0 (0)
Multi-racial	7 (7)
Asian/White	1 (1)
Hispanic/White	1 (1)
NA/White	3 (3)
NA/Black/White	1 (1)
NA/Black/Hispanic/White	1 (1)

*Self-reported

Supplementary Table 2. Human collagen type II (COL2A1) and IgG1 peptides used in ELISA with homologous murine collagen type II epitopes for arthritogenic antibodies

Name	Human Peptide (citrulline, B)	Human Peptide (native)	Murine Epitope (clone)*
COL2A1-558	GA**B**GLTG**B**PGDAG	GA**R**GLTG**R**PGDAG	A**R**GLT**GR**PGDA (C1^III)
COL2A1-693	GLVGP**B**GE**B**GFPGE**B**G	GLVGP**R**GE**R**GFPGE**R**G	LVGP**R**GERGFP (U1)
COL2A1-751	MPGE**B**GAAGIAGPKGD	MPGE**R**GAAGIAGPKGD	MPGE**R**GAAGIAGPK (J1)
COL2A1-885	GA**B**GAQGPPGATGFP	GA**R**GAQGPPGATGFP	A**R**GAQGPPGATGFP (D3)
COL2A1-976	LAGQ**B**GIVGLPGQ**B**	LAGQ**R**GIVGLPGQ**R**	LAGQ**R**GIV (E/F10)
IgG1-131	KDTLMIS**B**TPEV		
IgG1-167	VHNAKTKP**B**EEQYNSTY**B**VVSV		
IgG1-219	ISKAKGQP**B**EPQVYTLPPS**B**DEL		
IgG1-236	PS**B**DELTKNQVSLT		
IgG1-289	LYSKLTVDKS**B**WQQGNVFS		

* Antibodies that bind these murine COL2A1 epitopes in triple-helical form are arthritogenic in mice [1-3].

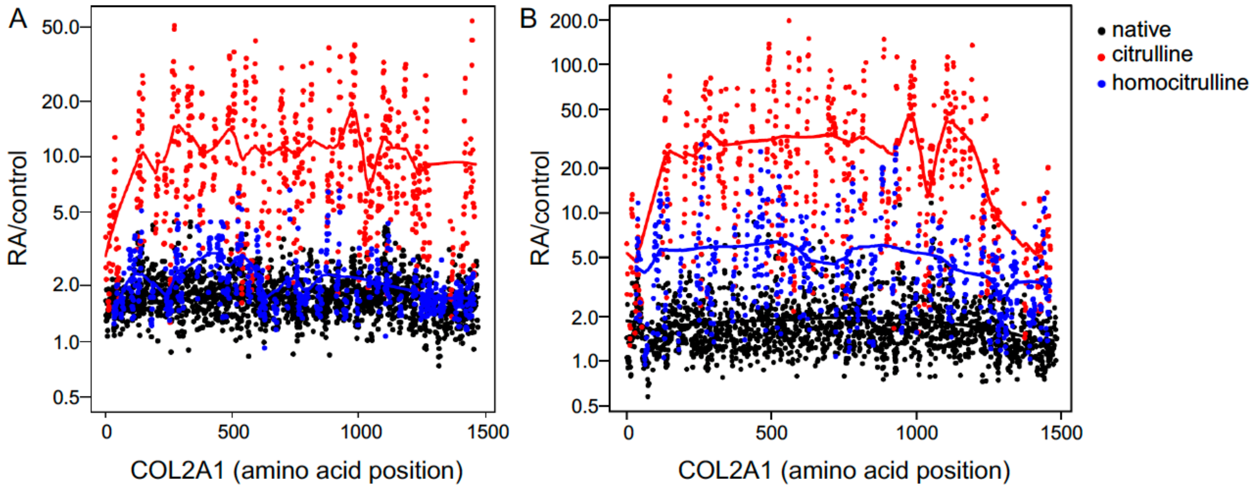

Supplementary Figure 1. Serum IgG from seropositive rheumatoid arthritis (RA) binds many linear citrulline-containing peptides derived from type II collagen. IgG binding values to all possible 12 amino acid linear peptides from collagen type II α1 (COL2A1, Uniprot P02458) using data from two previously published high density peptide arrays with sera from seropositive RA (anti-CCP and RF >2x the upper limit of normal) or control (no known autoimmune or inflammatory disease) participants [4,5] was used to generate fold value plots depicting each peptide. Peptides were present in three forms: native (black), all arginines replaced by citrullines (red), and all lysines replaced by homocitrullines (blue). A. Experiment 1 (n=12). B. Experiment 2 (n=8).

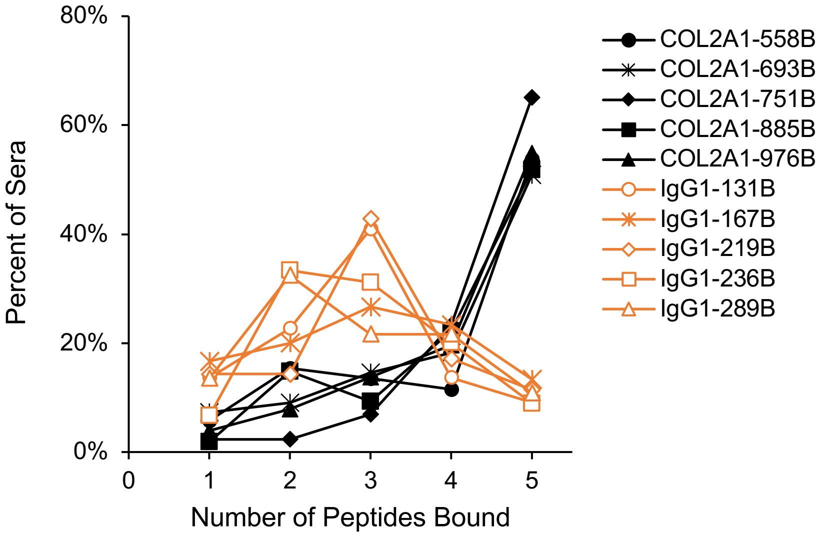

Supplementary Figure 2. IgG multi-reactivity for sera that contain each antibody. Among sera positive for IgG that binds each citrullinated collagen or IgG1 peptide (COL2A1-558B: n=52, COL2A1-693B: n=55, COL2A1-751B: n=43, COL2A1-885B: n=54, COL2A1-976B: n=51, IgG1-131B: n=44, IgG1-167B: n=30, IgG1-219B: n=35, IgG1-236B: n=45, IgG1-289B: n=37), the percent of sera with reactivity to one, two, three, four, or five collagen or IgG peptides, respectively, was quantified and graphed. Antibodies that bind to each peptide have different shapes and colors with black for collagen peptides and orange for IgG1 peptides.

Supplementary Table 3. Associations of HLA alleles in linkage disequilibrium with HLA-DRB1*04:01 and HLA-DQA1*01:02 with autoantibodies in rheumatoid arthritis *

		COL2A1						IgG1					
HLA **	n	558B	693B	751B	885B	976B	Multi	131B	167B	219B	236B	289B	Multi
**DRB1*04:01**	44	0.841	**0.044**	0.154	0.688	0.687	0.067	0.070	**0.015**	>0.999	0.228	0.534	**0.036**
DQA1*03:03	32	0.525	0.288	0.519	>0.999	0.832	0.127	0.829	0.640	0.074	0.288	0.078	>0.999
DQB1*03:01	36	>0.999	0.532	0.207	0.404	>0.999	0.089	0.406	0.822	0.132	0.297	0.285	0.827
DQB1*03:02	43	0.549	0.841	0.684	0.840	0.313	0.414	0.423	>0.999	0.833	0.547	0.531	0.394
**DQA1*01:02**	30	0.383	0.381	**0.029**	0.276	0.051	**0.044**	0.827	0.641	0.648	0.188	0.375	0.363
DRB1*11:01	3	0.606	0.587	0.257	0.094	0.114	0.273	>0.999	0.552	0.550	0.587	>0.999	0.549
DRB1*13:02	4	0.619	>0.999	0.312	0.622	0.618	0.299	0.629	0.581	>0.999	0.625	0.625	>0.999
DRB1*15:01	23	0.643	0.635	0.156	0.485	0.345	0.226	0.474	>0.999	0.804	0.341	0.325	0.456
DRB1*16:01	2	>0.999	0.500	>0.999	>0.999	0.495	>0.999	>0.999	0.512	>0.999	>0.999	>0.999	>0.999
DQB1*05:02	2	>0.999	0.500	>0.999	>0.999	0.495	>0.999	>0.999	0.512	>0.999	>0.999	>0.999	>0.999
DQB1*06:02	23	0.353	0.341	0.058	0.243	0.156	0.090	0.811	>0.999	0.804	0.341	0.325	0.456
DQB1*06:04	4	0.619	>0.999	0.312	0.622	0.618	0.299	0.629	0.581	>0.999	0.625	0.625	>0.999

* P values by Fisher's exact test that were <0.5 are depicted in green for a positive association and blue for a negative association. P < 0.05 in bold.

** HLA of interest in bold and HLA in linkage disequilibrium not in bold

Supplementary Table 4. Associations of partial HLA haplotypes containing HLA-DRB1*04:01 and HLA-DQA1*01:02 with autoantibodies in rheumatoid arthritis *

Partial Haplotype			COL2A1						IgG1					
HLA of interest	HLA in LD	n	558B	693B	751B	885B	976B	Multi	131B	167B	219B	236B	289B	Multi
DRB1*04:01	DQA1*03:03	25	0.653	0.248	0.488	>0.999	>0.999	0.238	0.364	0.218	0.230	0.489	0.095	0.234
	DQB1*03:01	25	>0.999	0.248	0.247	0.822	>0.999	0.238	0.173	0.218	0.230	0.248	0.095	0.096
	DQB1*03:02	29	0.665	**0.045**	0.181	>0.999	**0.047**	**0.045**	0.378	0.149	0.818	0.825	0.822	0.167
DQA1*01:02	DRB1*11:01	1	>0.999	0.450	>0.999	0.460	0.490	>0.999	>0.999	>0.999	>0.999	>0.999	>0.999	>0.999
	DRB1*13:02	4	0.619	>0.999	0.312	0.622	0.618	0.299	0.629	0.581	>0.999	0.625	0.625	>0.999
	DRB1*15:01	23	0.643	0.635	0.156	0.485	0.345	0.226	0.474	>0.999	0.804	0.341	0.325	0.456
	DRB1*16:01	2	>0.999	0.500	>0.999	>0.999	0.495	>0.999	>0.999	0.512	>0.999	>0.999	>0.999	>0.999
	DQB1*05:02	2	>0.999	0.500	>0.999	>0.999	0.495	>0.999	>0.999	0.512	>0.999	>0.999	>0.999	>0.999
	DQB1*06:02	23	0.353	0.341	0.058	0.243	0.156	0.090	0.811	>0.999	0.804	0.341	0.325	0.456
	DQB1*06:04	4	0.619	>0.999	0.312	0.622	0.618	0.299	0.629	0.581	>0.999	0.625	0.625	>0.999

* P values by Fisher's exact test that were <0.5 are depicted in green for a positive association and blue for a negative association. P < 0.05 is in bold and LD is linkage disequilibrium.

A

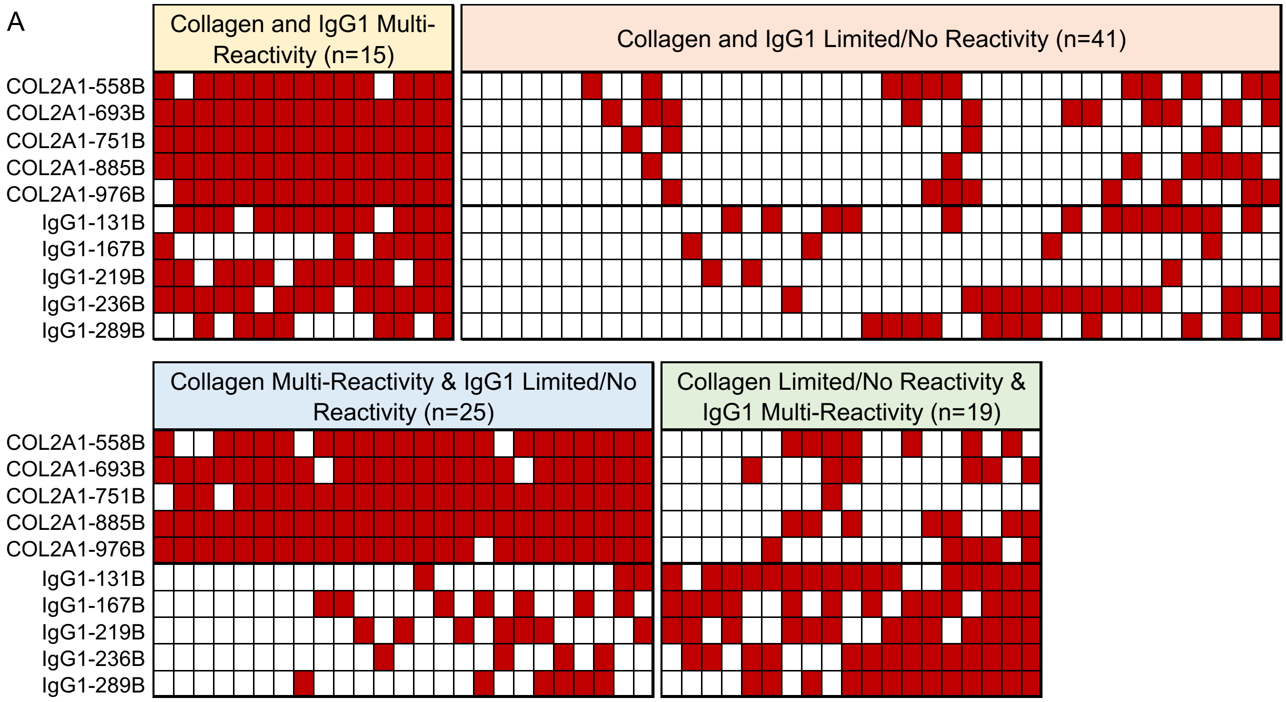

B

HLA-DRB1*04:01			Col(M)IgG(M) 6/15 (40%)
		Col(L)IgG(L) 17/41 (41%)	>0.999
	Col(M)IgG(L) 7/25 (28%)	0.304	0.498
Col(L)IgG(M) 14/19 (74%)	**0.006**	**0.027**	0.080

HLA-DQA1*01:02			Col(M)IgG(M) 5/15 (33%)
		Col(L)IgG(L) 10/41 (24%)	0.514
	Col(M)IgG(L) 12/25 (48%)	0.062	0.512
Col(L)IgG(M) 3/19 (16%)	0.052	0.522	0.417

Supplementary Figure 3. Four Group Analysis. A. Participants (n=100) were divided into four groups: serum multi-reactivity (M) with collagen peptides (IgG reactivity with 4-5 peptides) and IgG1 peptides (IgG reactivity with 3-5 peptides), limited or no reactivity (L) for collagen or IgG1 peptides, multi-reactivity with collagen peptides only, and multi-reactivity with IgG1 peptides only. B. The presence of each HLA allele was compared pairwise by Fisher's exact test for all groups with results depicted for HLA-DRB1*04:01 and HLA-DQA1*01:02, including the number and percent of subjects with the allele for each group in the colored boxes and p values in the white boxes. P < 0.05 in bold.

References for Supplementary Tables and Figures:

1 Bajtner, E., Nandakumar, K. S., Engstrom, A. & Holmdahl, R. Chronic development of collagen-induced arthritis is associated with arthritogenic antibodies against specific epitopes on type II collagen. *Arthritis Res Ther* **7**, R1148-1157, doi:10.1186/ar1800 (2005).

2 Schulte, S. *et al.* Arthritis-related B cell epitopes in collagen II are conformation-dependent and sterically privileged in accessible sites of cartilage collagen fibrils. *J Biol Chem* **273**, 1551-1561, doi:10.1074/jbc.273.3.1551 (1998).

3 Burkhardt, H. *et al.* Epitope-specific recognition of type II collagen by rheumatoid arthritis antibodies is shared with recognition by antibodies that are arthritogenic in collagen-induced arthritis in the mouse. *Arthritis Rheum* **46**, 2339-2348, doi:10.1002/art.10472 (2002).

4 Zheng, Z. *et al.* Disordered Antigens and Epitope Overlap Between Anti-Citrullinated Protein Antibodies and Rheumatoid Factor in Rheumatoid Arthritis. *Arthritis Rheumatol* **72**, 262-272, doi:10.1002/art.41074 (2020).

5 Bashar, S. J. *et al.* Limited Biomarker Potential for IgG Autoantibodies Reactive to Linear Epitopes in Systemic Lupus Erythematosus or Spondyloarthropathy. *Antibodies (Basel)* **13**, doi:10.3390/antib13040087 (2024).
